# PEP-1-CAT-Transduced Mesenchymal Stem Cells Acquire an Enhanced Viability and Promote Ischemia-Induced Angiogenesis

**DOI:** 10.1371/journal.pone.0052537

**Published:** 2012-12-28

**Authors:** Lei Zhang, Xiao-Wei Dong, Jia-Ning Wang, Jun-Ming Tang, Jian-Ye Yang, Ling-Yun Guo, Fei Zheng, Xia Kong, Yong-Zhang Huang, Shi-You Chen

**Affiliations:** 1 Institute of Clinical Medicine and Department of Cardiology, Renmin Hospital, Hubei University of Medicine, Shiyan, Hubei, People’s Republic of China; 2 Key Lab of Human Embryonic Stem Cell of Hubei Province, Hubei University of Medicine, Hubei, People’s Republic of China; 3 Department of Physiology, Hubei University of Medicine, Hubei, People’s Republic of China; 4 Department of Physiology & Pharmacology, The University of Georgia, Athens, Georgia, United States of America; Thomas Jefferson University, United States of America

## Abstract

**Objective:**

Poor survival of mesenchymal stem cells (MSC) compromised the efficacy of stem cell therapy for ischemic diseases. The aim of this study is to investigate the role of PEP-1-CAT transduction in MSC survival and its effect on ischemia-induced angiogenesis.

**Methods:**

MSC apoptosis was evaluated by DAPI staining and quantified by Annexin V and PI double staining and Flow Cytometry. Malondialdehyde (MDA) content, lactate dehydrogenase (LDH) release, and Superoxide Dismutase (SOD) activities were simultaneously measured. MSC mitochondrial membrane potential was analyzed with JC-1 staining. MSC survival in rat muscles with gender-mismatched transplantation of the MSC after lower limb ischemia was assessed by detecting SRY expression. MSC apoptosis in ischemic area was determined by TUNEL assay. The effect of PEP-1-CAT-transduced MSC on angiogenesis *in vivo* was determined in the lower limb ischemia model.

**Results:**

PEP-1-CAT transduction decreased MSC apoptosis rate while down-regulating MDA content and blocking LDH release as compared to the treatment with H_2_O_2_ or CAT. However, SOD activity was up-regulated in PEP-1-CAT-transduced cells. Consistent with its effect on MSC apoptosis, PEP-1-CAT restored H_2_O_2_-attenuated mitochondrial membrane potential. Mechanistically, PEP-1-CAT blocked H_2_O_2_-induced down-regulation of PI3K/Akt activity, an essential signaling pathway regulating MSC apoptosis. *In vivo*, the viability of MSC implanted into ischemic area in lower limb ischemia rat model was increased by four-fold when transduced with PEP-1-CAT. Importantly, PEP-1-CAT-transduced MSC significantly enhanced ischemia-induced angiogenesis by up-regulating VEGF expression.

**Conclusions:**

PEP-1-CAT-transduction was able to increase MSC viability by regulating PI3K/Akt activity, which stimulated ischemia-induced angiogenesis.

## Introduction

Ischemic diseases such as myocardial ischemia, cerebral ischemia, and lower limb ischemia are a major threat to human health. Stem cell therapy appears to be an effective treatment for the ischemic diseases. Stem cells transplanted into damaged tissues can differentiate into various functional cells including the cells that form blood vessels [1], which replace or repair the damaged tissue, improve blood supply [2], and reduce apoptosis and necrosis caused by ischemia and hypoxia.

Mesenchymal stem cells (MSC) are bone marrow-derived cells with multi-differentiation potential. Under certain conditions, MSC can differentiate into cardiomyocytes, adipocytes, osteoblasts, and neural cells, etc. [3]. Since MSC are easy to collect with a high transient or stable transfection efficiency of exogenous genes and a low immunogenicity, they are considered to be ideal progenitor cells for cell transplantation. A large number of animal and clinical trials have shown that MSC transplantation is safe and effective in the treatment of ischemic diseases. The survival rate of MSC transplanted into the ischemic region, however, is very low. It is reported that nearly 99% of MSC were lost after 24 h of transplantation [4]. Therefore, improving the survival rate of transplanted MSC is one of the critical issues for stem cell-based therapy.

Cells in ischemia or hypoxia environment produce a massive amount of reactive oxygen species (ROS). Accumulation of ROS causes cell apoptosis and necrosis, leading to tissue damage. ROS including superoxide anion (O^2−^•), hydroxyl radical (OH •), and hydrogen peroxide (H_2_O_2_), etc. damage tissues by activating a series of signaling pathways. Thus, suppression or elimination of excessive ROS generation is likely an effective strategy to improve the survival rate of transplanted MSC. Catalase (CAT) is a common enzyme found in nearly all living organisms exposed to oxygen. It catalyzes the decomposition of hydrogen peroxide to water and oxygen. It is a very important enzyme in biological defense system. Therefore, we expect that the survival rate of transplanted MSC will be improved through CAT transduction into MSC.

Our previous studies have confirmed that cell-penetrating peptide PEP-1 can efficiently transfer a variety of different biologically active proteins into cells [5]–[7]. In the present study, by using rat primary culture of MSC and H_2_O_2_-induced oxidative stress model [8], we found that PEP-1-CAT efficiently protected MSC against oxidative stress damage by activating PI3K/Akt signal pathways. *In vivo*, PEP-1-CAT transduction enhanced MSC tolerance to engrafted oxidative stress-induced injury and improved their viability in ischemic areas. Importantly, PEP-1-CAT-transduced MSC also enhanced angiogenesis by up-regulating VEGF expression.

## Materials and Methods

All animal studies are carried out in strict accordance with the recommendations in the Guide for the Care and Use of Laboratory Animals of the National Institutes of Health. The animal use protocol was approved by the Institutional Animal Care and Use Committee of Hubei University of Medicine.

### Isolation, Culture, and Phenotype Characterization of MSC

Bone marrow was ﬂushed from tibias and femurs of male donor mice. Bone marrow cells were cultured at 1×10^6^/cm^2^ in Dulbecco’s modified Eagle’s medium (DMEM,Invitrogen) with 5 g/L glucose supplemented with 20% (v/v) fetal bovine serum (FBS, Gibco, USA). The nonadherent cells were removed 24 h later by replacing the culture medium. Thereafter, half volume of the culture medium was replaced with fresh medium every 3 days until cell confluency. The monolayer MSC were expanded by splitting two times. MSC were characterized by Flow Cytometry. For osteoblast differentiation, the medium was replaced by osteogenic medium with 10^−7^ M dexamethasone, 0.2 mM ascorbic acid, and 10 mM β-glycerophosphate (Sigma). 21 day later when cell colonies displayed bone-like nodular aggregates of matrix mineralization, AgNO_3_ staining for calcium was used to visualize the mineral deposition. For adipocyte differentiation, the medium was replaced by adipocyte medium with 1 mM dexamethasone, 0.2 mM metacen, and 0.5 mM IBMX, 10 mg/ml insulin (Sigma). The medium was replaced every 3 days for 15 days. Adipocytes were visualized by Oil-O-Red staining for fatty drops.

For transplantation studies, MSC were labeled with cell tracker dye 1,1′-dioctadecyl-3,3,3′,3′-tetramethylindocarbocyanine perchlorate (DiI) by following the manufacturer’s instructions. The labeling efficiency was verified by Flow Cytometry.

### Transduction of CAT and PEP-1–CAT Fusion Proteins into MSC

CAT and PEP-1-CAT expression and purification were carried out as described previously [7]. Protein concentrations were measured by Bradford method [9]. MSC were grown to confluence on 75 cm^2^ flasks and pretreated with CAT or PEP-1-CAT at different dosage (0.5∼2.0 µM) for 1–24 h. The cells were then washed with phosphate-buffered saline (PBS) and treated with trypsin–EDTA followed by lysate preparation for western blot or enzyme activity assay. CAT activity was measured using CAT kit by following the manufacturer’s protocols (JianCheng Bioengineering Institute, China).

### Immunostaining

To directly visualize the transduction of CAT and PEP-1-CAT fusion protein into MSC, cells were treated with 2 µM of CAT or PEP-1-CAT. After 6 hrs of incubation, the cells were washed twice with 1×PBS and fixed with 4% paraformaldehyde for 15 min at room temperature. Immunocytochemistry was performed by incubation with specific primary antibody: rabbit anti-polyhistidine (diluted 1∶200) (Santa Cruz Biotechnology, USA) at 4°C overnight. Cells were then incubated with FITC-conjugated rat anti-rabbit Ig G (diluted 1∶250) at 25°C for 2 h. Nuclei were stained with DAPI (Sigma, USA).The immunoreactions were observed under a fluorescent microscope (Nikon, Japan). Angiogenesis in lower limb ischemic tissues was also detected by immunostaining using CD31 antibody.

### Induction of Oxidative Stress in MSC

MSC were cultured with medium containing 15% FBS or 2% FBS with or without CAT or PEP-1-CAT transduction. 6 h later, CAT or PEP-CAT-transduced MSC were treated with or without H_2_O_2_ (1 mM) for 1 h to induce oxidative stress. The supernatants and cells were then collected for further analysis.

### Annexin V and Propidium Iodide (PI) Binding Assay

To measure oxidative stress-induced apoptosis, MSC were pelleted, washed with PBS, and resuspended in 500 µl binding buffer (10 mM HEPES pH 7.4, 140 mM NaCl, 2.5 mM CaCl_2_)/1×10^6/^L cells. Cells were then incubated with Annexin V for 3 min followed by PI (Bender MedSystems, Austria) for 15 min. The apoptosis rate was evaluated by Flow Cytometry.

### Measurement of Malondialdehyde (MDA) Content, Lactate Dehydrogenase (LDH) Release, and Superoxide Dismutase (SOD) Activity

MDA, an end product of peroxidation of cell membrane lipids caused by oxidative free radicals, is considered as a reliable marker of oxidative damage [10]. MDA content was determined by measuring chromogen generation from the reaction of MDA with 2-thiobarbituric acid. LDH level is an indicator of cellular injury. SOD plays an important role in clearing O^2−^•, thereby protecting cells from oxidative damage. The LDH release and SOD activity were measured using commercial kits (JianCheng Bioengineering Institute, China).

### Measurement of Mitochondrial Membrane Potential

Mitochondrial transmembrane potential was assessed using a sensitive fluorescent probe 5,50,6,60-tetrachloro-1,10,3,30-tetraethyl-benzamidazolocarbocyanin iodide (JC-1, Invitrogen). Red emission from the dye is attributed to a potential-dependent aggregation of JC-1 in the mitochondria. Green fluorescence reflects the monomeric form of JC-1, appearing in the cytoplasm after mitochondrial membrane depolarization. Cells were grown on a 24-well plate and transduced with CAT or PEP-1-CAT followed by H_2_O_2_ treatment. Cells were then incubated with 5 mM JC-1 dye diluted in culture medium at 37°C for 15 min. Cells were washed three times with PBS and analyzed immediately using a fluorescence microscope (Nikon, Japan).

### Transduction of PEP-1-CAT in Rat Lower Limb with Ischemia Injury

240–280 g female Sprague-Dawley rats were obtained from the Experiment Animal Center at Hubei University of Medicine and housed at an appropriate temperature (25°C) and relative humidity (55%) with a fixed 12-hr light/dark cycle and free access to food and water. The animals were anesthetized with 10% chlorali hydras (250 mg/kg, i.p.), and the left femoral artery was ligated with 6-0 nylon suture. Surgery was performed under sterile conditions. 36 rats were randomly divided into two groups: one with transplantation of MSC and the other with transplantation of PEP-1-CAT-transduced MSC. One hour after the ischemia, the MSC or PEP-1-CAT-transduced MSC were injected at the surgical site. After one, three and seven days later, gastrocnemius tissues in the left legs were collected. 6 rats were collected for each time point. Apoptosis of the grafted MSC was detected using a TUNEL assay kit by following the manufacturer’s instruction (Beyotime). Briefly, tissues were incubated with 50 µl TUNEL test solution in dark at 37°C for 60 min, and then washed twice with PBS. The MSC apoptosis ratio was observed using fluorescent microscopy.

### Quantitative Reverse Transcription Polymerase Chain Reaction (qRT-PCR)

Total RNA from rat muscles was extracted using TRIZOL Reagent (Invitrogen). RNA concentration was determined by UV spectrophotometry. qRT-PCR was performed using THUNDERBIRD SYBR Master Mix (TOYOBO, Japan). Primer sequences were as follows: rSRY: 5′-GCA TTT ATG GTG TGG TCC CG-3′ (forward), 5′-TCT GTG TAG GGT CTT CAG TCT C-3′(reverse); β-actin: 5′-CGT TGA CAT CCG TAA AGA CCT C-3′ (forward), 5′-TAG GAG CCA GGG CAG TAA TCT-3′(reverse). qRT-PCR was performed on a Real-time PCR Detection System (Slan, Hongshi) with following cycles: 95°C for 1 min, followed by 95°C for 15 s, 58°C for 15 s, and 72°C for 45 s for 40 cycles. β-actin expression was used as an internal control.

### Western Blot

Cells or muscle tissues were lysed in RIPA buffer. 30 µg of proteins were separated in a 12% SDS-PAGE gel and transferred onto a nitrocellulose membrane (Millipore). The membrane was rinsed in Tris-buffered saline (TBS) with 0.1% Tween-20 (TBST) and blocked with 5% fat free milk in TBS at room temperature for 1 h. The membrane was then incubated with anti-VEGF, anti-Akt, anti-Akt-p, or anti-α-tubulin antibody (Sigma, 1∶5000) followed by incubation with horseradish peroxidase-conjugated secondary antibodies (1∶10000 dilution; Santa Cruz). The immunoblots were detected by enhanced chemiluminescence reaction (Amersham Pharmacia Biotech) and measured with densitometry.

### Statistical Analysis

Results from three independent experiments were expressed as mean ± S.D. Comparisons of parameters between two groups were made by unpaired t-test. Comparisons of parameters among more than two groups were made by one-way analysis of variance. P values <0.05 were considered statistically significant.

## Results

### Phenotype Characterization of MSC

**Figure 1:**
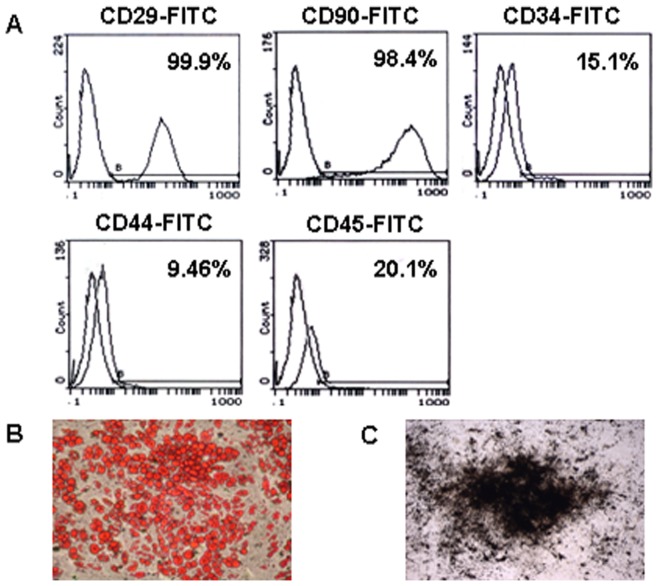
Characterization of MSC. (A) Phenotype characterization of MSC. Flow Cytometry analysis showed that almost all the cultured MSC expressed CD29 (98.4%) and CD90 (99.9%) while a small portion of MSC expressed CD34 (15.1%), CD44 (9.46%), and CD45 (20.1%). (B–C) Multipotential differentiation of MSC. MSC differentiation to adipocytes was shown by Oil-O-Red staining (B); and Osteoblast differentiation was detected by AgNO_3_ staining (C).

To characterize the phenotype of the isolated MSC, expression of MSC surface marker was analyzed. Almost all the cultured MSC expressed CD29 and CD90 although only a small portion of the cells expressed CD34, CD44 and CD45 (Fig. 1A). These characteristics were similar to what was previously described for MSC [11]. The ability of MSC in differentiation into osteocytes and adipocytes was confirmed in all cultures from various donors (Fig. 1B and 1C).

### Transduction of CAT or PEP-1-CAT into MSC

**Figure 2:**
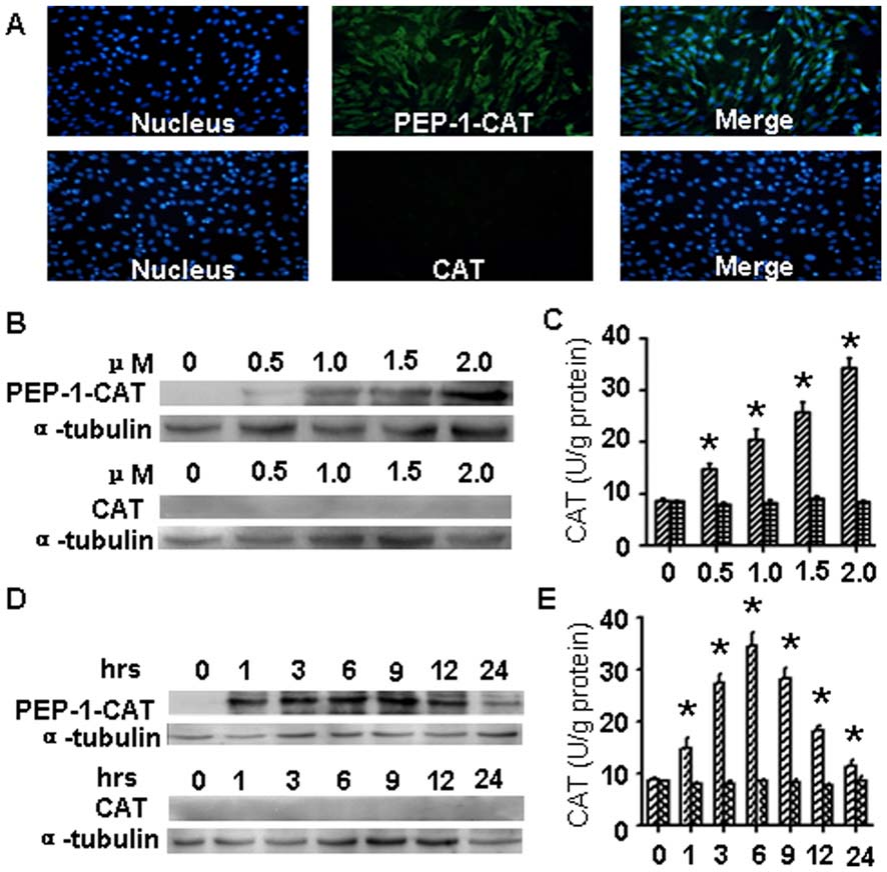
PEP-1-CAT, but not CAT, was transduced into MSC. (A) Transduction of PEP-1-CAT or CAT into MSC was detected by His tag antibody. Nuclei (Blue) were stained by DAPI. (B) Dose-dependent transduction of PEP-1-CAT and CAT was detected by western blot. (C) Dose-dependent catalase activity of PEP-1-CAT or CAT transduced into MSC. *P<0.05 compared to MSC without PEP-1-CAT (0 µM) (n = 3). (D) Time-dependent transduction of PEP-1-CAT and CAT was detected by western blot. (E) Time-dependent catalase activity of PEP-1-CAT or CAT transduced into MSC. *P<0.05 compared to MSC without PEP-1-CAT transduction (0 h) (n = 3).

His-tagged CAT and PEP-1-CAT were successfully expressed and purified. Their enzyme activities were 3.35×10^3^ U/g and 3.12×10^3 ^U/g, respectively, suggesting that PEP-1-CAT had similar enzymatic activities as the wild type CAT. Subcellular transduction of CAT or PEP-1-CAT fusion protein into MSC cells was confirmed by immunostaining using anti-His tag antibody. As shown in Fig. 2A, almost all cultured cells were transduced with PEP-1-CAT fusion protein. However, the green fluorescent signals were not detected in cells treated with CAT. To further investigate the transduction efficiency of PEP-1-CAT and CAT, we incubated different dosage of PEP-1-CAT or CAT with MSC for 1–6 h. As shown in Fig. 2B, incubation of 2 µM PEP-1-CAT enabled a sufficient transduction of PEP-1-CAT. CAT itself, however, was unable to transduce into MSC. To observe the time-dependent effect of PEP-1-CAT or CAT transduction, we incubated MSC with 2 µM PEP-1-CAT or CAT fusion protein in cell culture medium at different time interval, and analyzed CAT protein levels by western blotting. A high level of CAT protein in PEP-1-CAT-transduced cells was detected in MSC within 1 h of the transduction, and the protein level was gradually increased until 12 h after the transduction (Fig. 2D). Importantly, the transduced PEP-1-CAT had catalase activity, corresponding to its protein level (Fig. 2C and 2E).

### PEP-1-CAT Inhibited MSC Apoptosis While Decreased LDH Release, MDA Content and SOD Activity

**Figure 3:**
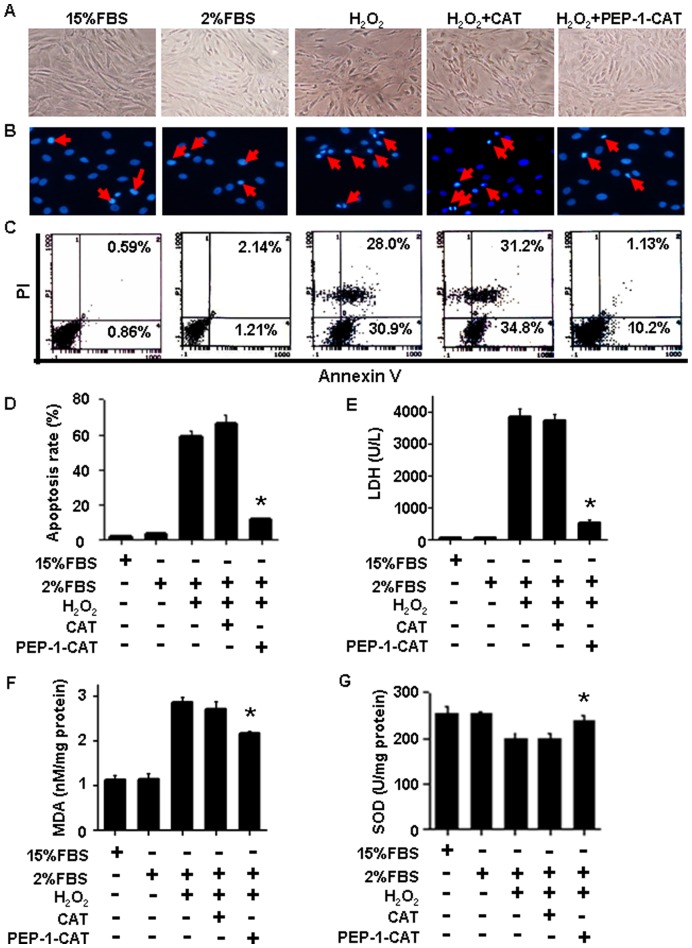
PEP-1-CAT inhibited H_2_O_2_-induced MSC apoptosis. (A) PEP-1-CAT restored oxidative stress-induced alteration of MSC morphology. (B) PEP-1-CAT restored the oxidative stress-induced DNA damage in nuclei as shown by DAPI staining. Red arrow indicates apoptotic cells. (C–D) MSC apoptosis due to oxidative stress was analyzed by Flow Cytometry. PEP-1-CAT blocked H_2_O_2_-incued apoptosis. *P<0.05 compared to the MSC treated with H_2_O_2_ or CAT (n = 3). (E) PEP-1-CAT inhibited LDH release in H_2_O_2_-treated MSC. *P<0.05 compared to the MSC treated with H_2_O_2_ or CAT (n = 3). (F) PEP-1-CAT inhibited MDA content in H_2_O_2_-treated MSC. *P<0.05 compared to the MSC treated with H_2_O_2_ or CAT (n = 3). (G) PEP-1-CAT transduction restored SOD activity in H_2_O_2_-treated MSC. *P<0.05 compared to the MSC treated with H_2_O_2_ or CAT (n = 3).

Spindle-shaped MSC were well organized under normoxia condition (15% FBS and 2% FBS group). H_2_O_2_ treatment caused cell shrinkage and distorted the overall appearance of MSC morphology (Fig. 3A) along with condensed nuclei, indicative of DNA damage (Fig. 3B). PEP-1-CAT transduction, however, restored the MSC cell morphology (Fig. 3A and 3B). Apoptosis was minimal when MSC were cultured in normoxia environment. H_2_O_2_ treatment caused 58.9% of MSC to undergo apoptosis. PEP-1-CAT transduction, however, decreased the apoptosis rate to 11.33% (Fig. 3C and 3D). CAT transduction did not reduce the H_2_O_2_-induced apoptosis (Fig. 3C and 3D).

LDH is an important enzyme involved in energy metabolism. LDH releases into blood circulation when tissue undergoes necrosis. Thus, LDH level indicates cellular injury. Compared to cells cultured in normoxia condition, H_2_O_2_ treatment induced a high level of LDH release, PEP-1-CAT transduction, however, prevented H_2_O_2_-induced LDH release (Fig. 3E), suggesting a protective role of PEP-1-CAT in MSC injury.

MDA, a lipid peroxidation metabolite, is positively correlated with free radicals under ischemia and hypoxia environments. MDA level reflects oxidative damage. MDA level was markedly increased due to oxidative stress injury, but decreased by PEP-1-CAT pretreatment (Fig. 3F). In contrast, SOD activity in PEP-1-CAT- pretreated cells were increased as compared to H_2_O_2_ or CAT-treated cells (Fig. 3G). These results demonstrate that PEP-1-CAT protects MSC from ROS damage through regulating the LDH, MDA and SOD levels.

### PEP-1-CAT Preserved Mitochondrial Membrane Potential in H_2_O_2_-treated MSC

**Figure 4:**
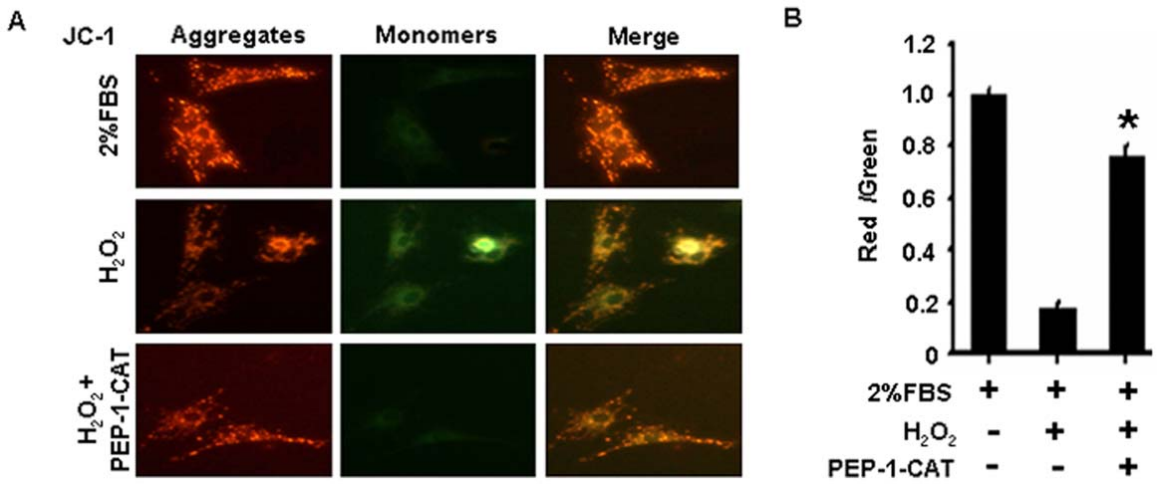
PEP-1-CAT prevented MSC from H_2_O_2_-induced decrease of mitochondrial membrane potential. (A) MSC were treated with 2% FBS, H_2_O_2_ with or without PEP-1-CAT. Mitochondrial membrane potential was measured by loading JC-1 onto the MSC and observed with a fluorescent microscope. Magnification: 200×. (B) Quantitatification of membrane potential. *P<0.05 compared to the MSC treated with H_2_O_2_ (n = 3).

JC-1 selectively enters mitochondria and reversibly changes its color from red to green as the membrane potential decreases [12]. MSC under normoxia conditions exhibited a normal mitochondria membrane potential as indicated by red ﬂuorescence staining of JC-1 (Fig. 4). H_2_O_2_ treatment caused formation of monomeric JC-1 in green-stained mitochondria, indicative of a loss of membrane potential (Fig. 4). PEP-1-CAT pretreatment, however, attenuated the H_2_O_2_-induced formation of JC-1 monomers (Fig. 4), suggesting that PEP-1-CAT protects MSC from apoptosis by inhibiting H_2_O_2_-induced reduction of mitochondria membrane potential.

### PEP-1-CAT Reversed H_2_O_2_-induced Decrease of Mitochondria Membrane Potential by Restoring PI3K/Akt Signaling

**Figure 5:**
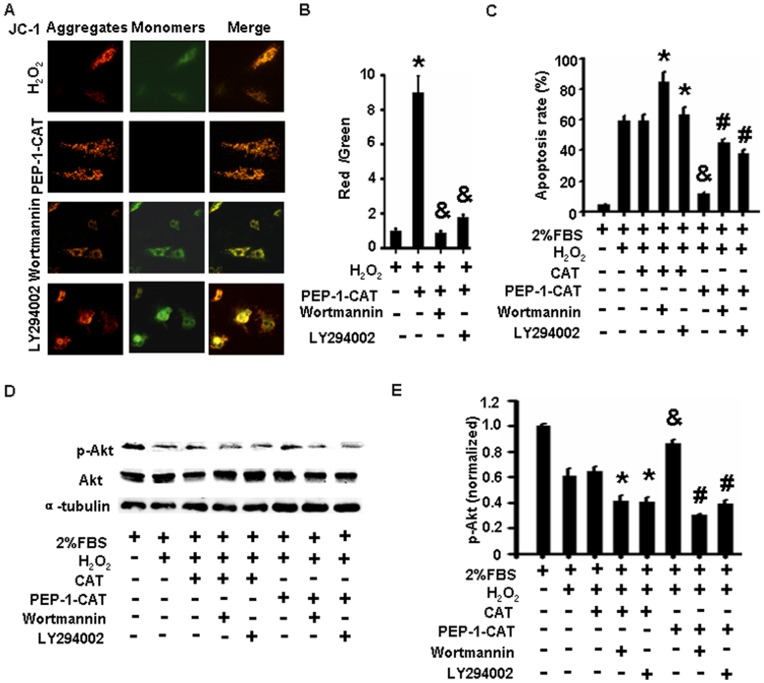
PEP-1-CAT prevented MSC from H_2_O_2_-induced decrease of mitochondria membrane potential through restoring PI3K/Akt signaling. (A) PI3K kinase inhibitor wortmannin or LY294002 blocked PEP-1-CAT-stimulated increase of the mitochondria membrane potential. (B) Quantitative assay results of membrane potential in (A). *P<0.05 compared to the MSC treated with H_2_O_2_. ^&^P<0.05 compared to the MSC pretreated with PEP-1-CAT (n = 3). (C) Wortmannin or LY294002 blocked PEP-1-CAT-mediated reduction of MSC apoptosis. *P<0.05 compared to CAT-transduced MSC without wortmannin or LY294002; ^&^P<0.05 compared to H_2_O_2_ or CAT-treated cells; ^#^P<0.05 compared to PEP-1-CAT-transduced MSC without wortmannin or LY294002 (n = 3). (D) PEP-1-CAT restored Akt phosphorylation (p-Akt) in MSC blocked by H_2_O_2_. Western blotting was performed to detect the expression of p-Akt and total Akt. α-tubulin served as an internal control. (E) Quantitative analysis of Akt phosphorylation by normalized to the total Akt level. *P<0.05 compared to CAT-transduced MSC without wortmannin or LY294002; ^&^P<0.05 compared to H_2_O_2_ or CAT-treated cells; ^#^P<0.05 compared to PEP-1-CAT-transduced MSC without wortmannin or LY294002 (n = 3).

Apoptosis is mediated by numerous apoptotic regulators including Bcl-2, Bax, caspases, p16, p53 and several other pathways such as p38 and PI3K/Akt signaling. To determine the factors that mediated PEP-1-CAT effect on mitochondria membrane potential, we examined the expression of these apoptotic regulators and the activation of p38 and PI3K/Akt signalings. We found that PI3K/Akt signaling, but not other proteins or signaling pathways (data not shown), played a critical role in mediating PEP-1-CAT function in restoring mitochondria membrane potential because blockade of PI3K signaling with specific inhibitor wortmannin or LY294002 significantly blocked PEP-1-CAT-enhanced MSC mitochondria membrane potential (Fig. 5A and 5B) while increased PEP-1-CAT-attenuated apoptosis (Fig. 5C). Consistent with its function in MSC apoptosis, PEP-1-CAT enhanced Akt activity as indicated by the increased Akt phosphorylation compared to the reduced Akt phosphorylation in H_2_O_2_-treated MSC (Fig. 5D and 5E). These data demonstrate that PEP-1-CAT inhibited oxidative stress-induced apoptosis by activating PI3K/Akt signaling pathway.

### PEP-1-CAT Improved the Survival of Grafted MSC in Ischemic Tissues and Promoted Angiogenesis

**Figure 6:**
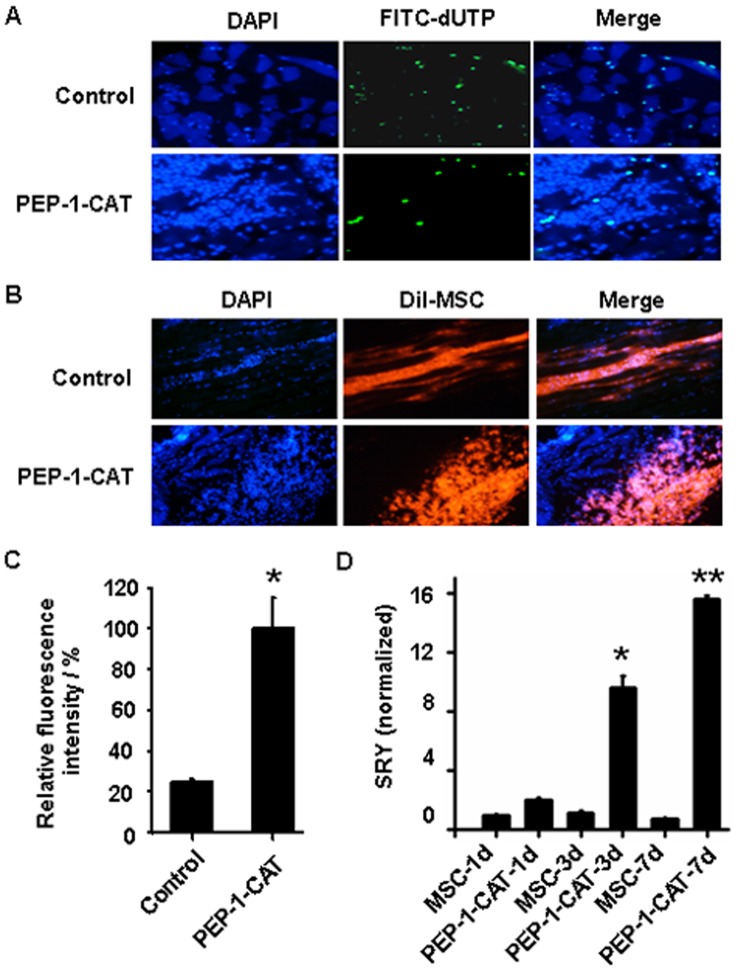
PEP-1-CAT enhanced MSC survival when transplanted into the ischemic lower limb of rat. (A) Apoptosis of MSC transplanted into the ischemic tissues was detected by TUNEL assay. Green fluorescence indicates apoptotic cells. DAPI stains nuclei (blue color). (B) Survival of grafted DiI-labeled MSC in the ischemic tissues was observed by fluorescence microscopy. Graft MSC show red color. DAPI stains nuclei (blue). (C) Quantitative analysis of DiI-labeled MSC. *P<0.05 compared to control (n = 3). (D) SRY expression was detected by qRT-PCR. *P<0.05, **P<0.001 compared to the graft of MSC without PEP-1-CAT transduction in its corresponding time points (n = 3).

To test if PEP-1-CAT transduced into MSC is biologically active *in vivo*, we measured the effect of PEP-1-CAT on the survival of grafted MSC in ischemic tissues with Tunnel assay, fluorescence imaging and sex determining region Y (SRY) gene expression in the rat leg muscles with ischemic injury. SRY gene is only expressed in the grafted male MSC from the donors. Compared to the control groups, less MSC apoptosis but a greater number of transplanted MSC (DiI-labeled cells) were observed in ischemic tissue with graft of PEP-1-CAT-transduced MSC (Fig. 6A, 6B and 6C). Moreover, transplantation of PEP-1-CAT-transduced male MSC led to a much higher expression of SRY gene in the ischemic tissue than the transplantation with control MSC (Fig. 6D), further demonstrating that PEP-1-CAT can effectively improve graft MSC survival in ischemic tissues.

**Figure 7:**
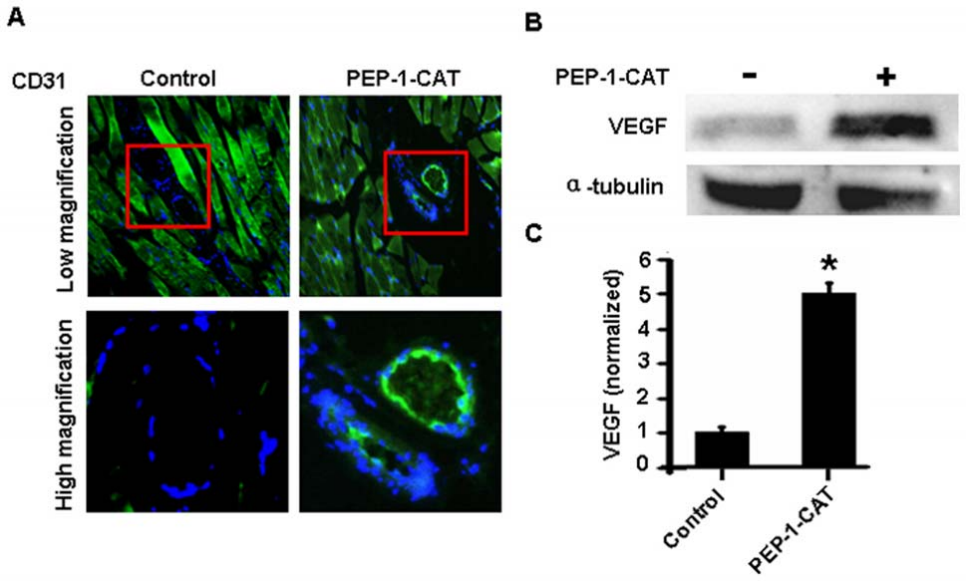
PEP-1-CAT-transduced MSC promoted ischemia-induced angiogenesis by upregulating VEGF expression. (A) CD31 expression in lower limb ischemic tissues was detected by immunostaining. (B) VEGF expression in lower limb ischemic tissues detected by western blot. (C) VEGF expression in lower limb ischemic tissues was normalized to α-tubulin. *P<0.05 compared to control (n = 3).

Most importantly, PEP-1-CAT-transduced MSC significantly improved ischemia-induced angiogenesis as shown by the increased CD31 expression and the newly formed blood vessel (Fig. 7A). To determine how PEP-1-CAT-contained MSC had promoted angiogenesis, we detected the expression of the angiogenesis regulator VEGF and found that VEGF expression was significantly upregulated for 4.9 fold in the ischemic tissue with graft of PEP-1-CAT-transduced MSC as compared to the cells without PEP-1-CAT (Fig. 7B and 7C). These data demonstrate that PEP-1-CAT-MSC stimulated angiogenesis via activating VEGF signaling.

## Discussion

Stem cell transplantation provides a new strategy for the treatment of ischemic diseases [13]. MSC, due to its multi-differentiation potential [14], low immunogenicity, easy access without ethic controversy, are considered to be ideal progenitor cells for cell transplantation. A large number of studies have shown that MSC transplantation is a very effective approach for treating ischemic diseases especially myocardial [15] and limb infarction [16]. Although MSC transplantation is safe and effective, MSC transplanted into the damaged area have a low vitality. Significant MSC death was observed within four days after grafting into the injured area largely due to ischemia [17]. To reduce MSC apoptosis rate, it is necessary to understand molecular mechanisms underlying the graft cell apoptosis in order to develop strategy for preventing the death of grafted cells [18]. Although different mechanisms such as host inﬂammatory response, loss of survival signal, proapoptotic or cytotoxic factors may contribute to death of grafted cells [17], [19]–[21], recent reports suggest that vitalities of graft cells in an ischemic environment are damaged by excessive ROS, which has limited the use of MSC transplantation.

Our study demonstrates that PEP-1-CAT transduction can increase the viability of MSC in ischemia environment by preventing MSC from oxidative stress-induced apoptosis. PEP-1-CAT inhibits MSC apoptosis by inhibiting LDH release and MDA content while enhancing SOD activity. In addition, PEP-1-CAT blocks oxidative stress-induced reduction of mitochondrial membrane potential, thus protects MSC membrane integrity.

Previous studies have shown that direct intramuscular injection of Akt-engineered MSC improved the function of infarct rat hearts [22], suggesting that Akt signaling may have played a role in improving MSC survival because Akt is a powerful survival signal in many systems [23]. However, the mechanism underlying Akt signaling in oxidative stress-induced apoptosis remain largely unknown. We found that H_2_O_2_ treatment leads to a downregulation of Akt phosphorylation in MSC. PEP-1-CAT transduction, however, reinstates the Akt activation to the normal level, suggesting that PEP-1-CAT enhances MSC survival via PI3K/Akt signaling pathway. Indeed, blocking PI3K/Akt signaling by specific inhibitor significantly attenuates PEP-1-CAT effect on inhibiting MSC apoptosis. Moreover, PI3K/Akt inhibitor blocks PEP-1-CAT function in restoring oxidative stress-induced reduction of mitochondrial membrane potential, suggesting that PI3K/Akt improves MSC survival under oxidative stress condition through regulating mitochondrial membrane potential.

PEP-1-CAT not only protects MSC from oxidative stress-induced apoptosis, but also has an effect on angiogenesis in ischemic tissues. PEP-1-CAT-transduced MSC significantly enhances angiogenesis as shown by the expression of endothelial cell marker CD31. PEP-1-CAT-contained MSC appear to induce angiogenesis by stimulating the production of VEGF. VEGF may be produced by MSC itself or MSC-derived cells. How PEP-1-CAT-transduced MSC stimulate VEGF expression will be an interesting subject for future study.

Taken together, our study has provided a novel approach to improve MSC survival in ischemic tissues, which may enhance the effectiveness of the stem cell therapy in ischemic diseases. Utilizing PEP-1-CAT transduced MSC for transplantation has two advantages. First, PEP-1-CAT can improve MSC survival, which facilitates the repair of the injured tissue. Second, PEP-1-CAT-transduced MSC enhance angiogenesis, which may accelerate the repair process.
